# The 20 kDa isoform of the human growth hormone variant alters adipose and muscle gene expression differently than human growth hormone

**DOI:** 10.1111/jne.70059

**Published:** 2025-06-16

**Authors:** Jonathan A. Young, Jolie Bogart, Mat Buchman, Silvana Duran‐Ortiz, Stephen Bell, John J. Kopchick, Darlene E. Berryman, Edward O. List

**Affiliations:** ^1^ Department of Biomedical Sciences Ohio University Heritage College of Osteopathic Medicine Athens Ohio USA; ^2^ Edison Biotechnology Institute Ohio University Heritage College of Osteopathic Medicine Athens Ohio USA; ^3^ Diabetes Institute Ohio University Heritage College of Osteopathic Medicine Athens Ohio USA

**Keywords:** GH‐2, GH‐V, growth hormone, growth hormone variant, placental growth hormone

## Abstract

The 20 kDa isoform of human growth hormone variant (20K hGH‐V) (derived from the GH2 gene) has previously been shown to promote growth but lacks the diabetogenic and lactogenic activities of human GH (derived from the GH1 gene). That is, 20K hGH‐V‐treated mice have similar body size and composition to hGH‐treated mice, as well as improved insulin sensitivity despite having similar adipose tissue mass. Furthermore, 20K hGH‐V‐treated prolactin receptor‐positive cancer cells exhibited significantly less growth compared to hGH treatment. The aim of this study was to use transcriptomics to compare the effects of 20K hGH‐V injection to that of hGH injection on adipose and muscle tissue. GH knockout (GHKO) mice, which do not produce endogenous GH, were injected with hGH or 20K hGH‐V daily for 5 days and dissected 4 h after the final injection. RNA was extracted from inguinal subcutaneous adipose tissue and quadriceps muscle and subjected to RNA sequencing. When comparing hGH to 20K hGH‐V, there were 73 genes that were significantly altered (*q* value <.05 and log_2_ fold change >1 or < −1) in adipose and 32 in muscle, with two genes (Cish and Sv2b) common to both tissues. Gene set enrichment analysis (GSEA) indicated that the adipose tissue of the 20K hGH‐V‐treated mice had decreased enrichment of genes associated with T and B lymphocytes compared to hGH‐treated adipose tissue. Furthermore, 20K hGH‐V treatment resulted in increased enrichment of genes associated with adipogenesis and carbon metabolism compared to hGH treatment. In muscle tissue, the electron transport chain and muscle contraction pathways were upregulated in 20K hGH‐V‐treatment, while cell cycle, extracellular matrix organization, and xenobiotic metabolism pathways were negatively enriched. While most genes and signalling pathways were similar between the two hormone treatments, the differentially expressed genes identified may help explain some of the phenotypic differences between 20K hGH‐V and hGH treatment and also suggest additional novel differences, notably muscle fibre type, immune cell infiltration, and fibrosis.

## 1 INTRODUCTION

Growth hormone (GH) has diverse growth and metabolic effects. GH stimulates longitudinal bone growth and increases muscle mass but also decreases adipose tissue mass. These effects of GH are demonstrated in clinical settings, as individuals with decreased GH action are characterized by short stature and obesity,^1^, ^2^ and recombinant GH treatment in GH‐deficient adults causes increased lean mass and decreased fat mass.^3^

There are other notable properties of hGH that could be viewed as deleterious. First, hGH is well known to be diabetogenic, as it inhibits insulin action. This diabetogenic effect was first demonstrated in animal studies by Nobel Laureate Bernardo Houssay in the 1930s^4^, ^5^ and in humans by Rabinowitz in the 1960s.^6^, ^7^ Accordingly, a major metabolic consequence of acromegaly is insulin resistance and, in some patients, diabetes.^8^ Second, hGH is not exclusive to the GH receptor, as it also binds and activates the prolactin receptor (PRLR).^9^ While the normal physiological role of GH's lactogenic activity is disputed, hGH has been shown to stimulate the growth of PRLR‐positive cancers such as breast,^10^, ^11^ prostate,^10^, ^11^, ^12^ and colon.^11^, ^13^, ^14^ Another side effect of GH is fibrosis, as GH stimulates excess extracellular matrix deposition in diverse tissues^15^, ^16^, ^17^ that can interfere with tissue function. These covert actions of GH are thought to be some of the reasons why excess GH reduces lifespan while removal of GH action increases lifespan in numerous ageing studies.^16^, ^18^, ^19^ Accordingly, the development of a GH molecule that retains the ability to regulate body growth and composition but lacks the less desirable actions might provide an improved therapeutic option for GH deficiency.

Humans have two separate GH genes: (1) GH‐N or ‘normal’ GH (also called *GH1* or pituitary GH) and (2) GH‐V or ‘variant’ GH (also called *GH2* or placental GH). GH‐N is produced in multiple isoforms at the protein level, with the 22 kDa isoform being the dominant isoform and the 20 kDa isoform the second most abundant.^20^ GH‐N is normally the only form of GH in humans except in pregnant females, where the placenta produces large amounts of GH‐V, which increases with pregnancy and eventually completely replaces pituitary GH in the third trimester. GH‐V, which likely resulted from gene duplication, only differs from GH‐N by 13 of 191 total amino acid residues.^21^, ^22^ Four isoforms of GH‐V have been described^23^ with the 22‐kDa isoform of GH‐V being the most abundant. Curiously, of the four GH‐V isoforms, isoform 4— which is the least abundant form of GH‐V, as it is only detected at the RNA level in extremely low levels, if at all^24^—has some remarkable properties when produced and given exogenously as a therapeutic drug.^22^, ^25^, ^26^ Specifically, 20K hGH‐V stimulates circulating IGF‐1 and promotes longitudinal bone growth, similar to hGH‐N in GH‐deficient mice.^25^ 20K hGH‐V also increases lean mass and decreases fat mass, similar to hGH‐N^25^ but lacks the diabetogenic^25^, ^26^ and lactogenic activities of hGH.^22^, ^26^

While different physiological outcomes in animals treated with human 20K hGH‐V and pituitary GH have been shown by two separate laboratories,^25^, ^26^ it is unclear what molecular mechanisms account for these differences. Accordingly, the current study uses RNA sequencing to capture the gene expression changes in muscle and adipose tissue collected from GH‐deficient mice treated with each hormone. Direct comparison between the two treatments (22K hGH‐N, which we refer to as ‘hGH’ throughout this paper, and 20K hGH‐V) allows the determination of the hGH effects that are altered or lacking in 20K hGH‐V.

## 2 MATERIALS AND METHODS

### 2.1 Animals

Male GH knockout (GHKO) mice^27^ at 4 months of age were used. Mice were given ad libitum access to water and ProLab RMH 300 chow and housed in a 14‐h light, 10‐h dark cycle. All animal procedures were approved by the Ohio University Institutional Animal Care and Use Committee and complied with federal, state, and local laws.

### 2.2 hGH/20K hGH‐V treatment

Recombinant human GH (hGH), the 20 kDa isoform of human GH variant (20K hGH‐V), or vehicle (0.4% NaHCO_3_) was injected subcutaneously daily for 5 days, as described previously.^25^ The dose used for both recombinant proteins was 5 μg/g body weight. Recombinant proteins were purchased from Protein Laboratories Rehovot (Cat. no. GHP‐24 and GHP‐12).

### 2.3 Body composition measurement

Body composition was determined before and after treatment using a Bruker Minispec NMR analyser (Bruker Corp., The Woodlands, TX) as described.^28^, ^29^

### 2.4 Dissection, blood collection, and RNA isolation

Mice were fasted for 12 h overnight and sacrificed 4 h after the final GH injection by placing them in CO_2_ until unconscious and bled from the orbital sinus before cervical dislocation as described.^30^, ^31^ GH injections were staggered by 8 min to ensure that all mice were dissected precisely 4 h after GH injection. Adipose tissue (inguinal subcutaneous) and muscle (quadriceps) were collected, flash frozen in liquid nitrogen, and held at −80°C until RNA extraction. RNA was extracted using QIAGEN (Hilden, Germany), RNeasy fibrous tissue mini kit Cat. # 74704 (muscle), or RNeasy lipid tissue mini kit Cat. # 74804 (adipose), according to the manufacturer's instructions.

### 2.5 Serum measurements

Insulin serum levels were measured using a Milliplex Mouse Metabolic Panel (MMHMAG‐44K; Millipore) and analysed using a Milliplex 200 Analyser (Millipore) according to the manufacturer's instructions as previously described.^32^, ^33^

### 2.6 RNA sequencing and differential gene expression analysis

Samples were prepared according to the library kit manufacturer's protocol, indexed, pooled, and sequenced on an Illumina HiSeq. Basecalls and demultiplexing were performed with Illumina's bcl2fastq software and a custom Python demultiplexing program with a maximum of one mismatch in the indexing read. RNAseq reads were then aligned to the Ensembl release 76 top‐level assembly with STAR version 2.0.4b.^34^ Gene counts were derived from the number of uniquely aligned unambiguous reads by Subread:featureCount version 1.4.5.^35^ Sequencing performance was assessed for the total number of aligned reads, the total number of uniquely aligned reads, and the features detected. The ribosomal fraction, known junction saturation, and read distribution over known gene models were quantified with RSeQC version 2.3.

All gene counts were then imported into the R/Bioconductor package EdgeR^36^ and TMM normalization size factors were calculated to adjust samples for differences in library size. Ribosomal genes and genes not expressed in three samples greater than one count per million in the n were excluded from further analysis. The TMM size factors and the matrix of counts were then imported into the R/Bioconductor package limma.^37^ Performance of the samples was assessed with Spearman correlations, a multi‐dimensional scaling plot, and hierarchical clustering. Weighted likelihoods based on the observed mean–variance relationship of every gene and sample were then calculated for all samples with the voomWithQualityWeights. The performance of all genes was assessed with plots of the residual standard deviation of every gene to their average log count with a robustly fitted trend line of the residuals. Differential expression analysis was then performed to analyse differences among conditions, and the results were filtered for only those genes with Benjamini‐Hochberg false‐discovery rate (FDR) adjusted *p*‐values less than or equal to .05.

### 2.7 PCA plot

To visualize the differences among samples, a principal component analysis (PCA) plot was constructed using R packages EdgeR,^36^ limma,^37^ and ggplot2.^38^ Genes with counts per million (CPM) ≥0.5 in ≥ 4 samples were used to generate the plot.

### 2.8 Enrichment Analysis

Gene set enrichment analysis (GSEA) was performed on the log_2_ fold change of all genes using the R package ClusterProfiler.^39^ KEGG,^40^ Reactome,^41^ and WikiPathways^42^ pathways were used in the analysis.

### 2.9 Alternate Gene Expression Profile Analysis

The gene expression profiles in Figures 4 and 6 were adapted from code published by Duncan et al^43^ and generated using the R package ComplexHeatmap.^44^

## 3 RESULTS

### 3.1 Body composition and insulin

**FIGURE 1:**
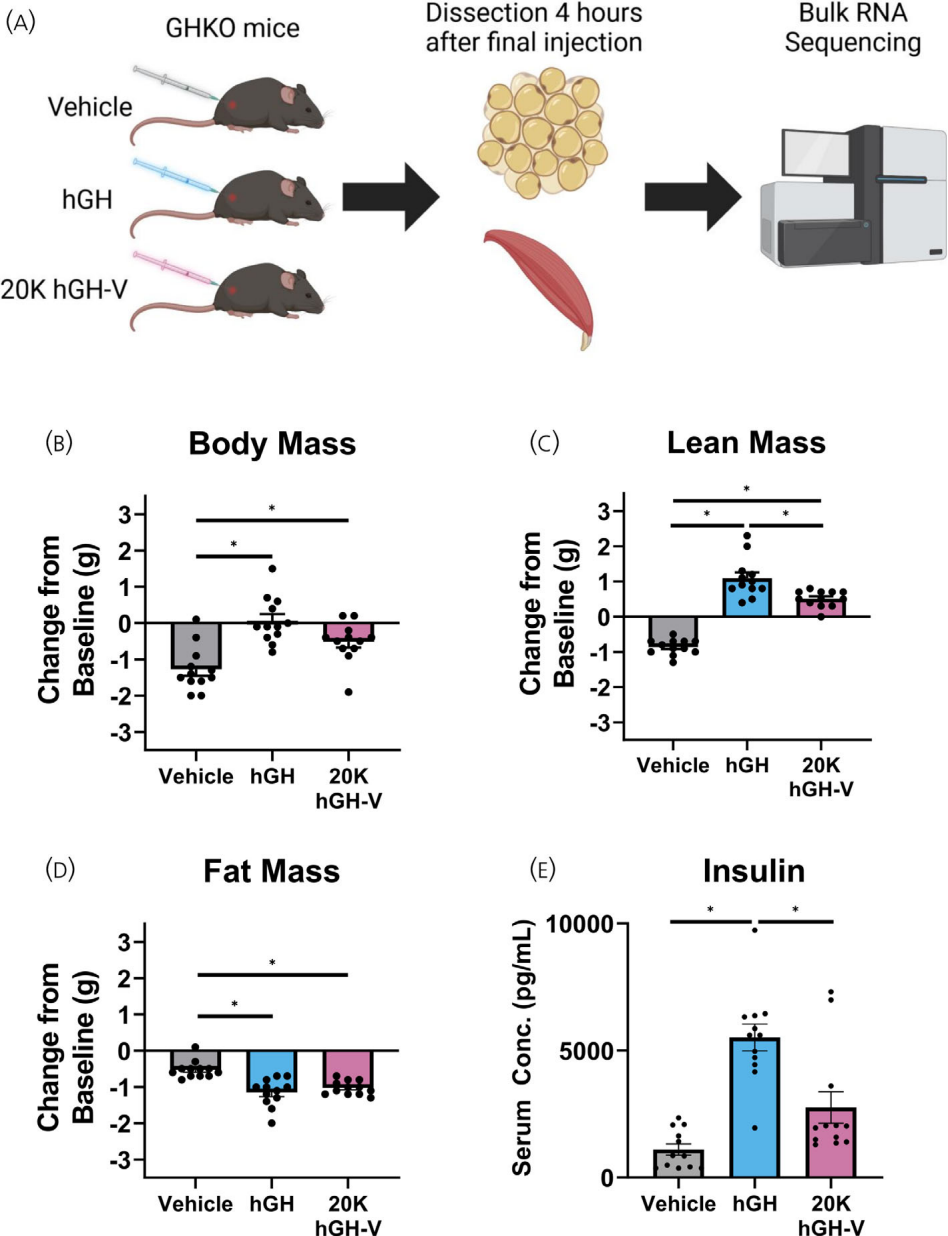
Experimental Design and Growth and Metabolic Parameters. A. Schematic of the experimental design. Mice were injected daily for 5 days with 5 μg/g body weight hGH, 20K hGH‐V, or vehicle control, and tissues were collected 4 h after the final injection and subjected to RNA sequencing. B. Total body mass of the mice shown as a change from baseline. C. Total lean mass of the mice shown as change from baseline, as determined by NMR analysis. D. Total fat mass of the mice shown as change from baseline, as determined by NMR analysis. E. Fasting insulin levels of the mice, after an overnight fast. * indicates one way ANOVA *p* < .05.

An outline of the study is shown in Figure 1A. Vehicle‐treated (control) mice lost over a gram of body mass during the short five‐day treatment, while hGH and 20K hGH‐V treated mice lost significantly less mass than controls (Figure 1B). However, there was no significant difference in lean mass between the hGH and 20K hGH‐V treatment. The 20K hGH‐V‐treated mice gained less lean mass than hGH‐treated mice, while controls lost lean mass (Figure 1C). As expected, hGH treatment caused a significant reduction in fat mass, a change that was matched by 20K hGH‐V treatment (Figure 1D). Fasting insulin levels were increased with hGH treatment compared to control, while 20K hGH‐V treatment did not significantly change insulin, although a trend towards increased insulin was present (Figure 1E).

### 3.2 Differentially expressed genes

**FIGURE 2:**
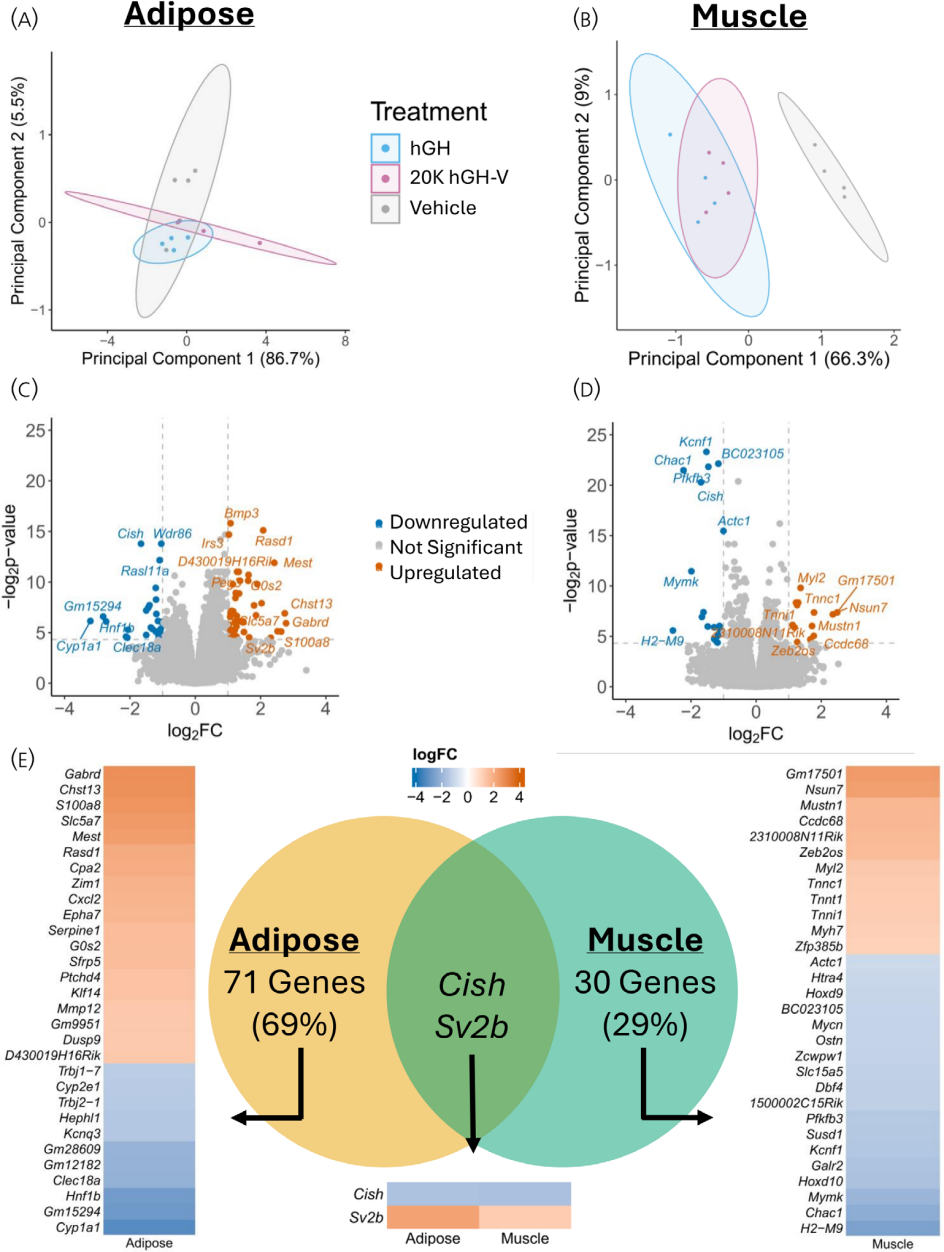
Differential Gene Expression. A, B. Principal components analysis of global gene expression in adipose and muscle tissue. C, D. Volcano plots showing log_2_ fold change and adjusted *p* value of all genes. Differentially expressed genes (genes with log_2_ fold change >1 or <−1 and adjusted *p* value <.05) are highlighted and labelled. E. Venn diagram and heatmaps of differentially expressed genes in each tissue. The Venn diagram shows that *Cish* and *Sv2b* are differentially expressed in both tissues. The heatmap shows the direction and magnitude of expression change.

PCA was used to compare global gene expression between hGH and 20K hGH‐V treatment in muscle and adipose (Figure 2A,B). There was no full separation between the treatment groups in either tissue, although adipose tissue showed more separation. These results were supported by the number of differentially expressed genes (DEGs, genes with log_2_ fold change >1 or <1 and adjusted *p* value <.05) in each tissue, as muscle had 32 DEGs and adipose had 73 DEGs (Figure 2C,D). Of these DEGs, only two (*Cish* and *Sv2b*) were differentially expressed in both tissues. The log_2_ fold change of the top 30 genes in each tissue is shown in heatmaps in Figure 2E.

### 3.3 Enrichment analysis—adipose

**FIGURE 3:**
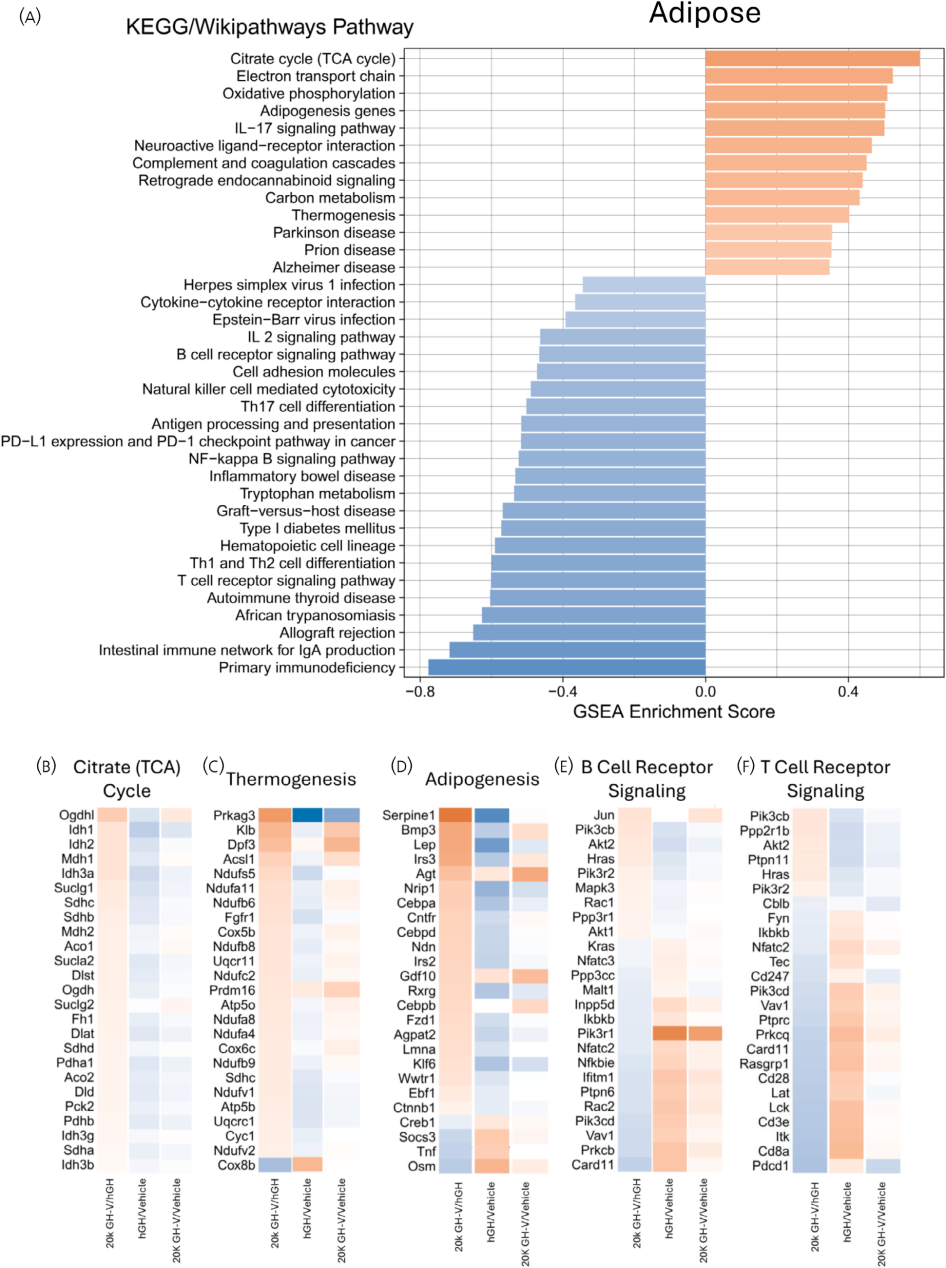
A. Adipose GSEA (gene set enrichment analysis). GSEA was performed on a list of all genes sorted by log_2_ fold change, and pathways that are significantly enriched at the top (positive enrichment score) or bottom (negative enrichment score) of the list in the adipose tissue are shown. B–F. Heatmaps of selected pathways of interest in adipose tissue. For each pathway, log_2_ fold changes of the top 20 most significant genes are shown in three different comparisons: 20K hGH‐V vs. hGH, 20K hGH‐V vs. vehicle, and hGH vs. vehicle.

To understand the biological function of the gene expression changes in each tissue, GSEA^45^ was performed using pathways from KEGG, WikiPathways, and Reactome. This analysis gives information not only about which pathways are altered but also an indication of whether these pathways are up‐ or downregulated. In the adipose tissue, 36 pathways were significantly enriched (Figure 3A), with 13 having positive scores, indicating that they are enriched among the genes with the highest positive fold changes. Also, 23 pathways had negative scores, indicating that they are enriched among the most negative fold changes.

The upregulated pathways comparing 20K hGH‐V to hGH include metabolic pathways such as the citrate (TCA) cycle (0.600 enrichment score [ES]), adipose tissue‐specific pathways such as adipogenesis (0.502 ES) and thermogenesis (0.402 ES), and neurodegenerative disease pathways such as Parkinson's disease (0.354 ES). Negatively enriched pathways include predominantly immune‐related pathways such as T cell receptor signalling (−0.602 ES) and B cell receptor signalling (−0.466 ES), though other pathways, such as tryptophan metabolism (−0.537 ES) and cell adhesion (−0.473 ES) are also downregulated.

Because the fold changes in the enrichment analysis compared 20K hGH‐V injected mice to hGH injected mice, the changes compared to saline injection are somewhat obscured, as a positive fold change could indicate that 20K hGH‐V increases the expression of that gene compared to saline but could also indicate that 20K hGH‐V decreases the gene expression less than hGH. To compare how each treatment differs from control, Figure 3B–F shows the fold change of the top 25 most significant genes in selected pathways using three comparisons: 20K hGH‐V/hGH, hGH/control, and 20K hGH‐V/control. For example, the TCA cycle is shown as upregulated in 20K hGH‐V/hGH, but when compared to vehicle, it shows that hGH downregulates many of the genes in the pathway compared to vehicle, while 20K hGH‐V also downregulates many of these genes, just to a lesser extent.

### 3.4 Alternate gene expression profile analysis—adipose

**FIGURE 4:**
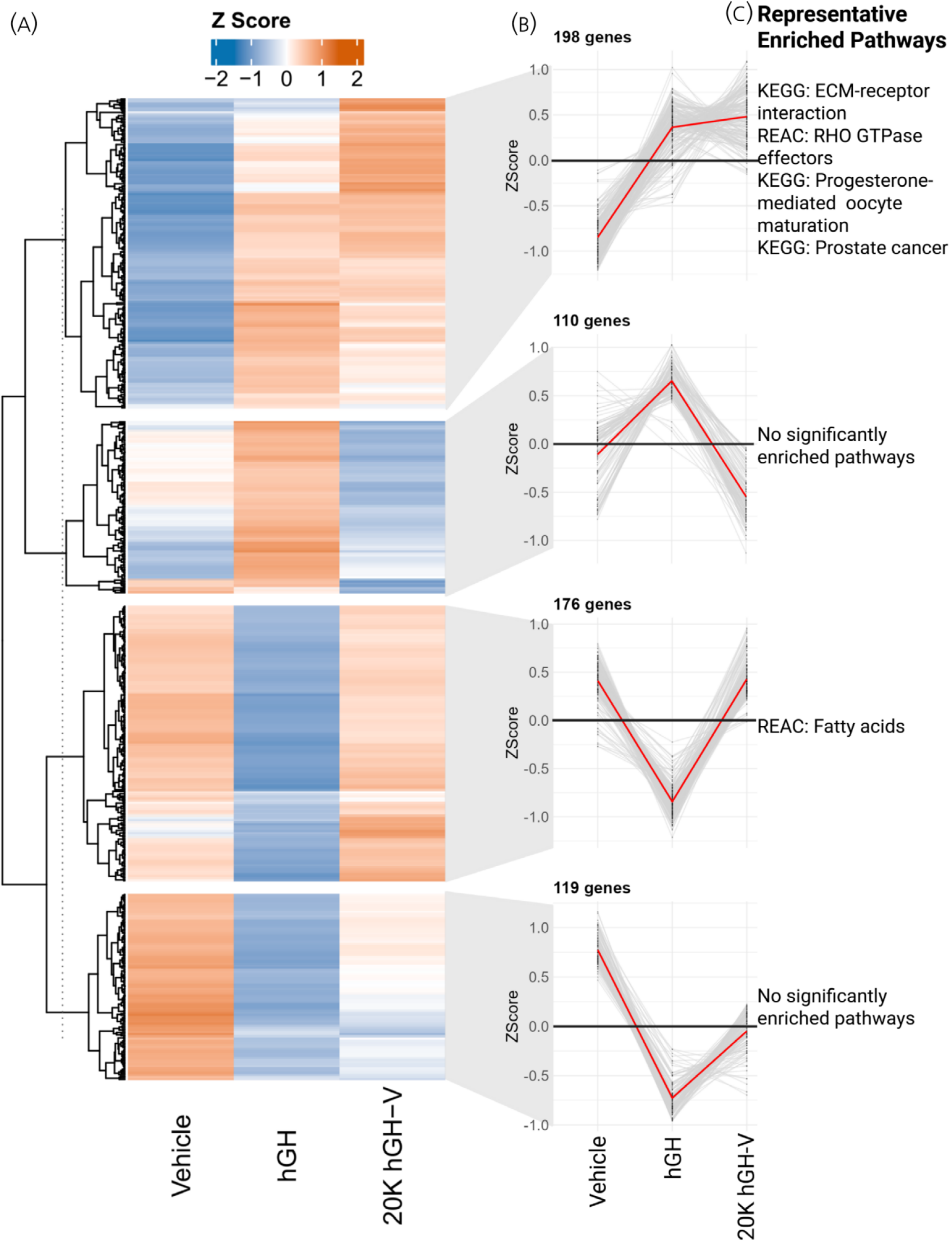
Alternate gene expression profile analysis of adipose tissue. A. Heatmap showing the expression Z score of each gene that was significantly altered in at least one of the pairwise comparisons between treatments, with genes clustered into four gene groups based on their expression pattern. B. Expression pattern of each gene group showing the average expression across treatments. C. Representative pathways enriched in the genes of each gene group.

To further assess differential gene expression among the saline, hGH, and 20K hGH‐V groups, we generated a gene expression profile highlighting genes significantly altered in at least one pairwise comparison (Figure 4A). These genes were categorized into four distinct expression pattern groups (Figure 4B), followed by over‐representation analysis (ORA) to identify enriched biological functions. Representative pathways associated with each gene group are shown in Figure 4C.

### 3.5 Enrichment analysis—muscle

**FIGURE 5:**
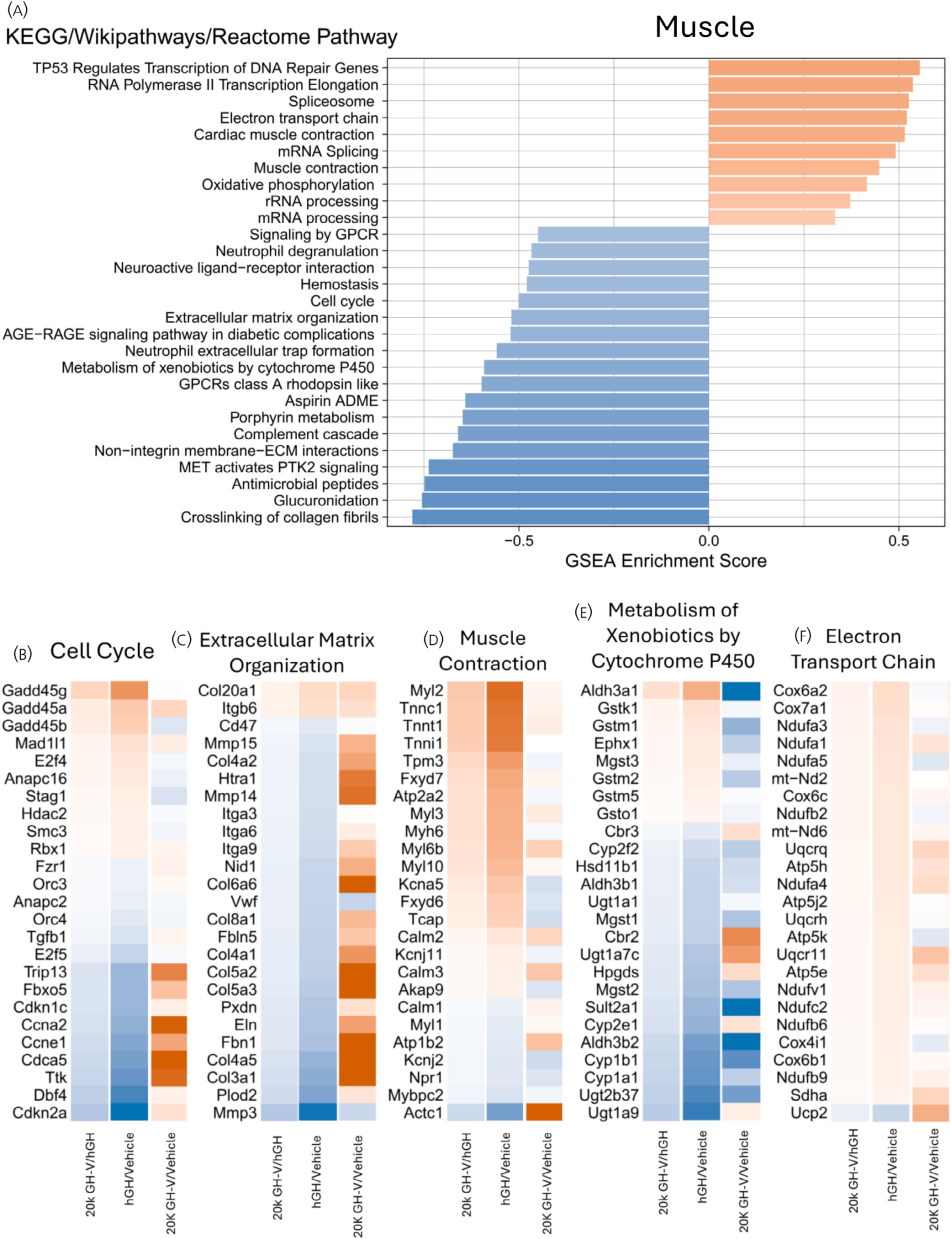
A. Muscle GSEA (gene set enrichment analysis). GSEA was performed on a list of all genes sorted by log_2_ fold change, and pathways that are significantly enriched at the top (positive enrichment score) or bottom (negative enrichment score) of the list in the muscle tissue are shown. B‐F. Heatmaps of selected pathways of interest in muscle tissue. For each pathway, log_2_ fold changes of the top 20 most significant genes are shown in three different comparisons: 20K hGH‐V vs. hGH, 20K hGH‐V vs. vehicle, and hGH vs. vehicle.

GSEA was also performed on the muscle results, and 28 pathways were significantly enriched, with ten positive (upregulated) and 18 negative (downregulated) pathways (Figure 5A). Positively enriched pathways include multiple transcription‐related as well as metabolic (oxidative phosphorylation [0.416 ES] and electron transport chain [0.521 ES]) pathways and muscle‐specific pathways (muscle contraction [0.448 ES]). Negatively enriched pathways include a diverse set of pathways. Altered pathways with a previously known link to GH signalling include extracellular matrix organization (−0.520 ES), cell cycle (−0.501 ES), and metabolism of xenobiotics by cytochrome p450 (−0.592 ES). Figure 5B–F shows the fold change of the top 25 most significant genes in selected pathways using three comparisons: 20K hGH‐V/hGH, hGH/control, and 20K hGH‐V/control. For example, the extracellular matrix organization pathway is downregulated in the 20K hGH‐V/hGH comparison, but comparing each hormone to vehicle demonstrates that both 20K hGH‐V and hGH increase expression of extracellular matrix genes, but hGH increases them more than 20K hGH‐V does.

### 3.6 Alternate Gene Expression Profile Analysis—Muscle

**FIGURE 6:**
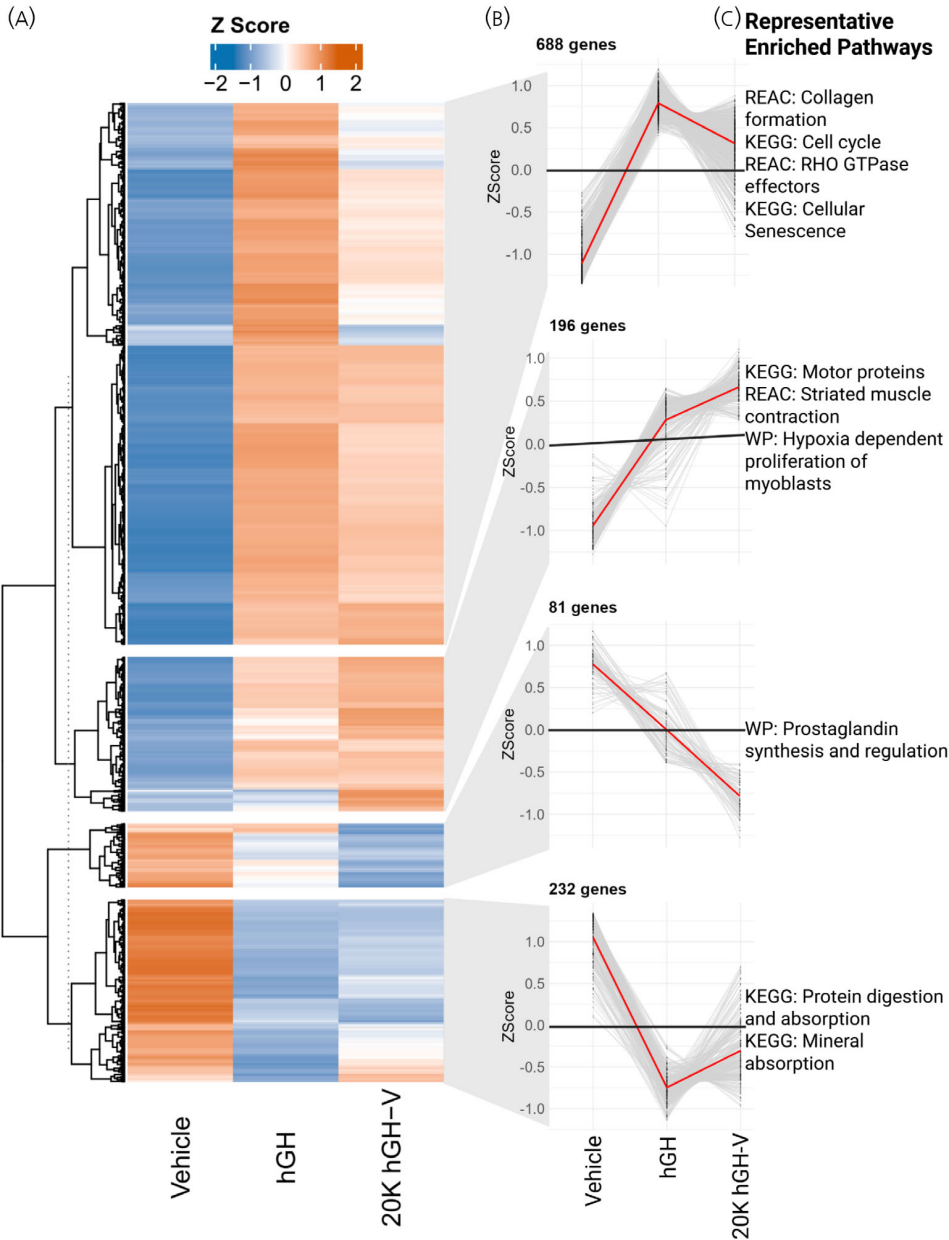
Alternate gene expression profile analysis of muscle tissue. A. Heatmap showing the expression Z score of each gene that was significantly altered in at least one of the pairwise comparisons between treatments, with genes clustered into four gene groups based on their expression pattern. B. Expression pattern of each gene group showing the average expression across treatments. C. Representative pathways enriched in the genes of each gene group.

Muscle gene expression was also assessed using the same method as adipose, in which we generated a gene expression profile highlighting genes significantly altered in at least one pairwise comparison (Figure 6A). These genes were categorized into four distinct expression pattern groups (Figure 6B), followed by ORA to identify enriched biological functions. Representative pathways associated with each gene group are shown in Figure 6C.

## 4 DISCUSSION

This is the first study to evaluate gene expression differences between treatments with 20K hGH‐V or hGH. Previous studies have shown that the 20K hGH‐V, a rare isoform of GH‐V, maintains some of the positive effects of hGH, increasing lean mass and decreasing fat mass, but lacks the diabetogenic and lactogenic activities of hGH.^22^, ^25^, ^26^ In this study (the first using adult mice), GHKO mice treated with hGH and 20K hGH‐V for 5 days lost less body mass than controls, despite losing more fat mass. 20K hGH‐V and hGH treatment resulted in similar losses in fat mass, but both treatments had increased lean mass compared to controls, with hGH increasing lean mass more than 20K hGH‐V. As expected, hGH treatment led to increased fasting insulin, demonstrating the diabetogenic action of hGH, while 20K hGH‐V did not significantly increase insulin levels. These results agree with previous reports about the properties of 20K hGH‐V and show that they persist in adult mice.^22^, ^25^, ^26^ Thus, these similar molecules have similar growth properties but differ in their metabolic properties. To further understand the differences between hGH and 20K hGH‐V, the transcriptomes of two major insulin‐sensitive tissues (adipose and muscle) were assessed after treatment with hGH, 20K hGH‐V, or vehicle. Because the circulating insulin levels differ between the treatment groups, it is unclear whether the gene expression changes are driven by insulin rather than directly by the treatment. To inspect global gene expression patterns, a PCA was performed. The PCA showed a better separation of hGH‐treated samples from 20K hGH‐V samples in adipose tissue than in muscle (Figure 1A,B). Indeed, when looking at the number of DEGs (Figure 1E), there is a higher number of significantly altered genes in adipose than in muscle. This indicates that hGH and 20K hGH‐V actions on gene expression differ more in adipose tissue than in muscle.

Two genes were significantly altered (log_2_ fold change >1 or <−1 and adjusted *p* value <.05) in both tissues, *Cish* and *Sv2b*. *Cish* is a SOCS (Suppressors of Cytokine Signalling) gene, is strongly induced by GH action,^46^ and serves as a negative feedback response to GH treatment. As the RNA in this experiment was collected 4 h after GH injection, the increase in *Cish* suggests that the GH‐stimulated pathways are being downregulated by the time of collection. In both tissues, when 20K hGH‐V was compared to hGH, *Cish* expression was decreased, indicating that 20K hGH‐V does not stimulate *Cish* expression to the same extent that hGH does and may have a different timing of target tissue response. The *Sv2b* gene encodes synaptic vesicle glycoprotein 2B, which is involved in exocytosis and vesicle trafficking in neurons and endocrine cells.^47^ *Sv2b* is not highly expressed in adipose or muscle tissue^48^ and has no reported function in either tissue, so the effect of increased *Sv2b* expression in 20K hGH‐V compared to hGH treatment is unclear. An interesting property of the data demonstrated by the volcano plots (Figure 2C,D) is that the volcano plot of muscle DEGs is tall, while the volcano plot for adipose is wide. This is caused by muscle having genes with low adjusted *p*‐values compared to the adipose results (more consistent changes), with adipose having genes with very large fold changes compared to the muscle results (larger changes but higher variance). Notably, 20K hGH‐V‐treated muscle reveals strongly downregulated genes compared to hGH treatment, which is caused by both 20K hGH‐V downregulating certain genes more than hGH (*Chac1* and *Pfkfb3*) and by 20K hGH‐V upregulating certain genes less than hGH (*BC023105*, *Kcnf1*, and *Actc1*).

### 4.1 20K hGH‐V vs. hGH comparison

To understand the pattern of genes altered by 20K hGH‐V compared to hGH, GSEA was performed on the RNAseq results. This analysis sorts all genes by fold change and determines which pathways are enriched at the top of the sorted gene list (upregulated) or at the bottom (downregulated). The adipose GSEA results are summarized in Figure 3. Upregulated pathways include multiple metabolic pathways such as the citrate cycle, electron transport chain, oxidative phosphorylation, and carbon metabolism. Another significantly upregulated pathway in the 20K hGH‐V to hGH comparison is adipogenesis. This result is interesting considering that adipose tissue mass was unchanged between 20K hGH‐V and hGH treatment, which could indicate that a longer treatment with 20K hGH‐V may show differences in adipose tissue mass due to differences in the anti‐adipogenic effects of the two treatments.

### 4.2 Treatments vs. saline comparison

By comparing the expression of these genes to vehicle treatment (Figure 4) we can see a broader picture of the relationship between treatments. We see that the direct comparison of 20K hGH‐V to hGH showed the pathways mentioned above (citrate cycle, electron transport chain, oxidative phosphorylation, carbon metabolism and adipogenesis) are upregulated. However, when each treatment is compared to vehicle controls, we see that both treatments actually downregulate these pathways, with 20K hGH‐V downregulation occurring to a significantly lesser extent.

Thermogenesis is another pathway that is upregulated in the 20K hGH‐V treatment group, but it differs in that the genes in this pathway are actually oppositely regulated when comparing 20K hGH‐V or hGH to vehicle controls. These results imply that 20K hGH‐V may induce beiging in adipose tissue, while hGH inhibits it. This differs from previous reports that indicate that GH action is positively correlated with adipose beiging in mice, except during chronic kidney disease‐induced cachexia.^49^, ^50^, ^51^, ^52^ The increased energy demand caused by the increased thermogenesis suggested by these data may help explain the relative increases in metabolic pathways in 20K hGH‐V compared to hGH.

In terms of the pathways that are downregulated in 20K hGH‐V‐treated adipose tissue, almost all of the significant pathways are immune system‐associated, including the B cell receptor signalling pathway, T cell receptor signalling pathway, and primary immunodeficiency, among many others. Once again, these pathways are downregulated in 20K hGH‐V compared to hGH, which is driven by the genes being upregulated compared to saline to a lesser extent in 20K hGH‐V than hGH. That is, these immune pathways are upregulated by 20K hGH compared to saline but not to the same extent that hGH upregulates them. These results hint at decreased immune cell infiltration into the adipose tissue, which is a sign of improved health of the adipose tissue. In previous studies, bGH transgenic mice, but not GHRKO mice, were found to have increased infiltration of macrophages and T helper cells in the subcutaneous WAT compared to WT mice,^53^, ^54^ confirming that excess GH results in immune cell infiltration of the adipose tissue, which may be decreased by replacing GH with 20K hGH‐V.

In the alternate gene expression profile analysis of the adipose tissue, the largest single group of genes had similar expression under hGH and 20K hGH‐V treatment, both being higher than the expression level when treated with the vehicle. ORA enrichment of these genes showed 16 significant pathways, including many extracellular matrix pathways as well as an RHO GTPase pathway, an oocyte maturation pathway, and a prostate cancer pathway, the latter two of which are perplexing to see in adipose tissue. The other three gene groups, making up the majority of genes, had differing expression between hGH and 20K hGH‐V, cementing the differing effects on the adipose tissue transcriptome despite their relative similarity in growth phenotype. The only one of these gene groups to have a significantly enriched pathway (fatty acids) was the gene group with 176 genes that were similarly expressed in vehicle and 20K hGH‐V treatment but lower in hGH treatment.

The upregulated pathways in muscle can be placed into three broad categories: transcription, oxidative phosphorylation, and muscle contraction. The metabolic pathways, electron transport chain, and oxidative phosphorylation are altered in a similar manner as the adipose tissue, meaning that, when compared to vehicle, 20K hGH‐V downregulates these genes less than hGH does.

The muscle contraction pathway is upregulated in 20K hGH‐V‐treated muscle compared to hGH‐treated muscle. This is somewhat unexpected considering that 20K hGH‐V did not increase lean mass as much as hGH. When examining the expression of muscle contraction genes compared to saline, hGH downregulates the expression of many of these genes while 20K hGH‐V does not, leading to a relative increase in 20K hGH‐V compared to hGH. Many of the most downregulated genes in hGH‐treated muscle are associated with slow‐twitch muscle fibres, indicating that hGH may induce a slow‐to‐fast transformation in the quadriceps muscle of GHKO mice while increasing muscle mass. The effects of GH on muscle fibre type in rodents are controversial.^55^ Some studies have shown that hypophysectomized rats without hGH treatment have increased slow‐twitch fibres,^56^, ^57^ which would be consistent with our results. However, this disagrees with a previous study that reported an increase in slow‐twitch fibres in hypophysectomized rats treated with hGH,^58^ while other studies showed no change in fibre type when hypophysectomized rats were treated with hGH.^57^, ^59^, ^60^ The effects of 20K hGH‐V add further complexity to this topic, as the data in this study indicate that hGH‐V does possess the same properties as hGH regarding muscle fibre type.

The list of downregulated pathways in muscle is diverse in terms of functions. However, some of these have previously been reported to be regulated by GH, including extracellular matrix and metabolism of xenobiotics by cytochrome P450. Our laboratory and others have previously reported^15^, ^16^, ^17^, ^61^, ^62^ that excess activation of the GH pathway is associated with fibrosis in diverse tissues. The results of this experiment indicate that 20K hGH‐V does not induce fibrotic genes to the same extent as hGH, which could be another advantageous property of 20K hGH‐V therapy compared to hGH therapy. Previous studies have also linked GH with xenobiotic metabolism, with *Ghr* gene‐disrupted mice showing increased expression of xenobiotic metabolism enzymes in the liver,^63^, ^64^ indicating an inverse relationship between GH and xenobiotic metabolism, especially in males.^65^, ^66^ These results indicate that, at least in the muscle, hGH increases expression of xenobiotic metabolism enzymes, while 20K hGH‐V downregulates many of the same enzymes. These results could be the result of a tissue difference in the regulation of xenobiotic metabolism, so the effects of 20K hGH‐V on the liver regulation of xenobiotic metabolism remain to be seen.

In the alternate gene expression profile analysis of the muscle, by far the largest single group of genes (688 genes) once again had similar expression under hGH and 20K hGH‐V treatment, both higher than the vehicle treatment expression. ORA enrichment of these genes showed many significant pathways, including a large number of extracellular matrix pathways as well as an RHO GTPase pathway, similar to what was seen in the adipose tissue. Other pathways enriched in this gene group are involved with the cell cycle/mitosis and cellular senescence. The expression pattern of the gene group with 196 genes showed a trend towards 20K hGH‐V treatment having the highest expression levels. There were three pathways enriched in this gene group, all associated with muscle function. The gene group that had the highest expression in vehicle treatment and the lowest in 20K hGH‐V treatment (81 genes) had a significant enrichment for the prostaglandin synthesis pathway, while the fourth gene group (highest in vehicle treatment and similar in hGH and 20K hGH‐V; 232 genes) had a significant enrichment in pathways for protein and mineral absorption. Overall, in the muscle tissue, hGH and 20K hGH‐V treatments had similar expression patterns for most of the significant genes, which is confirmed by the relatively low number of DEGs in the direct comparison of 20K hGH‐V and hGH shown in Figure 2E. This study compared injections of 20K hGH‐V to those of hGH in adult GHKO mice, and the physiological readouts confirmed much of the previous knowledge about the molecule, including its similar promotion of growth while lacking diabetogenic properties. However, by examining the transcriptional response of adipose and muscle tissue to short‐term 20K hGH‐V or hGH treatment, the acute response of these tissues to 20K hGH‐V and hGH was determined for the first time. This study uncovered some expected changes in metabolic pathways between the two types of GH; however, it also found significant differences in immune cell infiltration, fibrosis, and muscle fibre type that require further investigation.

## AUTHOR CONTRIBUTIONS

**Jonathan A. Young:** Conceptualization; methodology; software; data curation; formal analysis; writing – review and editing; writing – original draft; investigation; visualization. **Jolie Bogart:** Investigation. **Mat Buchman:** Investigation. **Silvana Duran‐Ortiz:** Investigation. **Stephen Bell:** Investigation. **John J. Kopchick:** Writing – review and editing; funding acquisition; project administration; conceptualization. **Darlene E. Berryman:** Funding acquisition; writing – review and editing; project administration; conceptualization. **Edward O. List:** Funding acquisition; writing – review and editing; project administration; supervision; conceptualization.

## CONFLICT OF INTEREST STATEMENT

The authors have no conflicts of interest to disclose.

## ETHICS STATEMENT

All animal procedures were approved by the Ohio University Institutional Animal Care and Use Committee and complied with federal, state, and local laws.

## Data Availability

The data that support the findings of this study are available from the corresponding author upon reasonable request.
